# Cell-free measurements of brightness of fluorescently labeled antibodies

**DOI:** 10.1038/srep41819

**Published:** 2017-02-02

**Authors:** Haiying Zhou, George Tourkakis, Dennis Shi, David M. Kim, Hairong Zhang, Tommy Du, William C. Eades, Mikhail Y. Berezin

**Affiliations:** 1 Department of Radiology, Washington University School of Medicine, St. Louis, MO 63110, USA; 2 Department of Medicine and Siteman Cancer Center Flow Cytometry Core, Washington University School of Medicine, St. Louis, MO 63110, USA.

## Abstract

Validation of imaging contrast agents, such as fluorescently labeled imaging antibodies, has been recognized as a critical challenge in clinical and preclinical studies. As the number of applications for imaging antibodies grows, these materials are increasingly being subjected to careful scrutiny. Antibody fluorescent brightness is one of the key parameters that is of critical importance. Direct measurements of the brightness with common spectroscopy methods are challenging, because the fluorescent properties of the imaging antibodies are highly sensitive to the methods of conjugation, degree of labeling, and contamination with free dyes. Traditional methods rely on cell-based assays that lack reproducibility and accuracy. In this manuscript, we present a novel and general approach for measuring the brightness using antibody-avid polystyrene beads and flow cytometry. As compared to a cell-based method, the described technique is rapid, quantitative, and highly reproducible. The proposed method requires less than ten microgram of sample and is applicable for optimizing synthetic conjugation procedures, testing commercial imaging antibodies, and performing high-throughput validation of conjugation procedures.

Validation of antibodies has been recognized as a critical challenge in clinical and preclinical studies^1^,^2^. The difficulty in antibody validation stems from the large variability of antibody hosts, methods of purification, and quality controls. Rigorous validation techniques include chromatography^3^, Western blotting^4^, overexpression of the antigen in cell lines with isotype control^5^, protein and tissue microarrays^6^, confocal microscopy to verify tissue and subcellular distribution^7^, knock-down phenotypes^5^, as well as surface plasmon resonance^8^, Raman spectroscopy^9^ and X-ray crystallization^10^. While these methods can adequately measure the affinity of the antibody to the target, they cannot assess its fluorescent properties independently from the target.

Quantification of the fluorescence brightness of the labelled antibody is important for the following reasons: i) it enables optimization of the conjugation protocol and selection of the fluorescent label to achieve the highest brightness, ii) provides quality control of the conjugates, leading to the consistency of the imaging results, iii) defines and minimizes the necessary dosage, lowering the toxicity of the imaging procedure while maintaining a sufficient signal-to-noise ratio.

The majority of fluorescent antibody applications in biochemical assays are based on a two-component assay, where a secondary antibody is labelled with a fluorescent tag^11^, such as a fluorescent dye, quantum dot, or an upconverting nanocrystal^12^,^13^. Relatively recently, a different class of imaging antibodies (ImAbs) carrying fluorescent tags for *in vivo* assays of small animals^14^, with potential use in humans^15^, has emerged. ImAbs are fluorescently labelled antibodies that identify an antigen of interest in live organisms primarily for imaging and diagnostic applications. ImAbs combine the specificity of the primary antibody with the reporting function of a secondary antibody.

Although ImAbs have relatively slow pharmacokinetics and low tissue permeability compared to smaller molecules, they have in many cases unmatched specificity. The wide variety of secondary and ImAbs requires their fluorescence measurements to be standardized and reported, but this information is rarely available. Instead it is often assumed that fluorescence brightness of the labelled antibody is equivalent to the brightness of the free dye, which is often incorrect^16^.

The major quantitative parameter describing fluorescence activity of a labelled antibody, and therefore its sensitivity, is the brightness (*B*), which is defined as the product of molar absorptivity of the dye (*ε*) at the wavelength of excitation (*λ*), quantum yield (*Q*) and the number of fluorescent tags (*N*_*f*_) per antibody, also known as degree of labelling, (DOL) according to eq. 1:


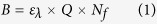


Direct measurement of brightness requires extensive optical characterization and relatively large amounts of sample that are generally unavailable or expensive. Knowing molar absorptivity factors of free dyes and their quantum yields is insufficient, as the change in the absorption and emission spectra due to H-type aggregation and quenching effects from nearby residues distorts the results by up to 90%, such as in quenchbodies. In quenchbodies, a single chain variable region of antibody labelled at the N-terminal region shows significant fluorescence quenching due to nearby tryptophan residues. Binding of the quenchbody to an antigen leads to the disruption of the quenching effect and fluorescence enhancement^17^. Calculations of DOL that rely on absorption properties are prone to large errors due to uncertainty about the molar absorptivity of the label on the conjugate. Finally, contamination of the conjugate with free dye occurs occasionally. The free dye has minimal effects on imaging results due to washing procedures or faster clearance, especially if the dye is hydrophilic, but might substantially mask optical characterization of the labelled antibody. Thus, quantitative evaluation of fluorescent antibodies remains a challenge and standardized methods have not yet been established.

Herein, we have developed an approach that enables rapid quantitative relative assessment of the fluorescence brightness of imaging antibodies. The primary innovation in our method is the use of beads instead of cells in combination with flow cytometry. The beads are in general uniform, not subject to the irregularities present in cell populations and cell-related artefacts and can be standardized.

## Results and Discussion

**Figure 1:**
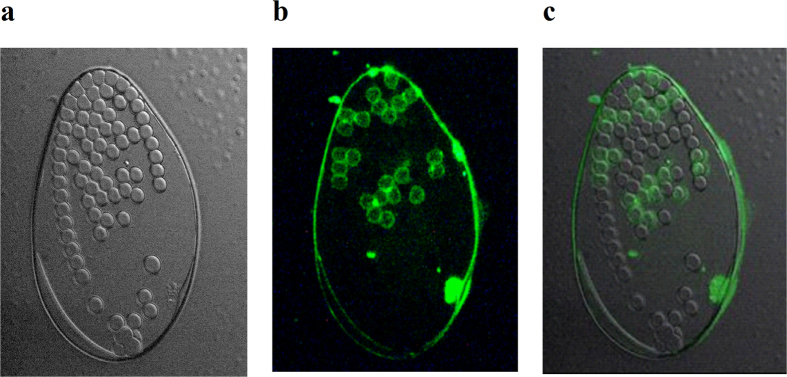
(**a**) Bright field (DIC) image of a drop of positive and negative beads treated with a fluorescently labelled antibody (Obj. 40X). (**b**) Fluorescent image of the same drop reveals two populations, with approximately half of the beads labelled, (**c**) overlap (see also Figure S1, Supplementary Information showing ca. 50% beads positive (labelled)).

We propose a rapid method to assess the fluorescence activity of labelled antibodies using a mix of polystyrene microspheres that are coated for high affinity to kappa chains (positive beads) and uncoated beads that do not bind antibodies (negative beads), but provide a measure of background fluorescence. These beads could be analysed by standard flow cytometry techniques, in which the signal intensity from individual beads is proportional to the fluorescent brightness of the antibody. This method is similar to calculating a Stain Index for assessing the brightness of a fluorophore via the relationship between the positive and the negative (background) signals using cells^18^,^19^. In the Stain Index (SI) measurements, the fluorophore conjugated to an antibody is tested in a cell culture. Flow cytometry results from analysing a mixture of stained (positive) and non-stained (negative) cells are used to calculate the SI. This index provides a good functional definition of reagent brightness and enables side-by-side comparison of the dye-antibody conjugates. However, a (article) cell-based approach requires standardized cells and identical clones of antibodies for each measurement that are not always possible to provide. In our method, a one-to-one mixture of positive and negative beads is added to a test tube containing the dye-antibody conjugates, after which the mixture is analysed by flow cytometry. The two components provide distinct positive and negative populations, which can be seen with a microscope (Fig. 1) or on a flow cytometry histogram (Figure S1, Supplementary Information).

The fluorescence intensity of the positive beads relative to that of the negative beads provides a quantitative but relative basis for antibody assessment. The separation between the populations at the beads’ saturation (*I*_*s*_) can be used as a measurable parameter directly related to fluorescence brightness (*B* ∝ I_*s*_) of the labelled antibody.

We envisioned that bimodal distribution of the histogram, in which the pixel intensities clustered around two reasonably separated values, might serve as a benchmark for brightness evaluation and a quantitative approach for antibody validation. Highly overlapped modes (such as monomodal distributions) suggest low brightness, while well-separated modes indicate strongly fluorescent probes. Thus, we first fitted a histogram with a two-member Gaussian model to obtain two populations. Identified parameters were then used to calculate the gap between the two peaks to provide what we called Peak Mean Distance (*I*). Other metrics, such as Stain Index (*SI*) and Bhattacharyya distance (*D*_*B*_) that takes into account standard deviations were also used. All metrics can be calculated using our developed in-house software, *Label-It*, based on MATLAB.

**Figure 2:**
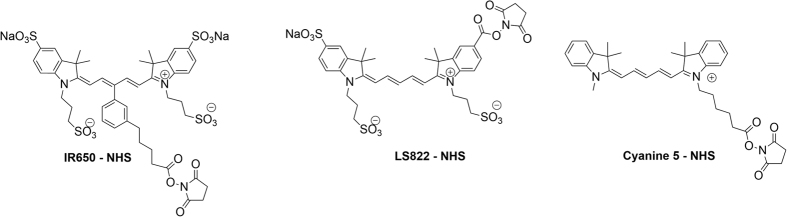
Fluorescent dyes used in this study for IgG labelling and flow cytometry testing.

The commercial dye IR650-NHS and one prepared in our lab, LS822-NHS, were selected for this study because of their high hydrophilicity (featuring four and three sulfonates, correspondingly) (Fig. 2). A hydrophobic dye with no sulfonate groups, Cyanine 5-NHS, was used to test the limitation of this method. All of the selected dyes absorbed and emitted in the same spectral range, allowing us to use identical settings on the flow cytometer (spectra of LS822 are in Figure S2, Supplementary Information). The dyes were conjugated to the antibody IgG through lysine residues using standard NHS chemistry^20^.

The physical stability of the beads upon treatment was confirmed by comparison of their scattering patterns (Figure S3, Supplementary Information). Scattering is quite sensitive to size of the particles and is commonly used in flow cytometry to determine the type of cells as well as the change in the cells’ morphology. To determine the percentage of beads that changed their physical shape upon treatment, we used the Overton cumulative subtraction algorithm^21^ available in *FlowJo*. Each sample was compared against control not-treated beads. We found the fractions of beads that changed their shape (judged by the changes in front scattering (FSC) and site scattering (SCS) distributions) were consistently less than 6%, with most cases less than 1%. This high similarity between two populations of beads, treated and non-treated, suggests the physical and chemical stability of the beads.

**Figure 3:**
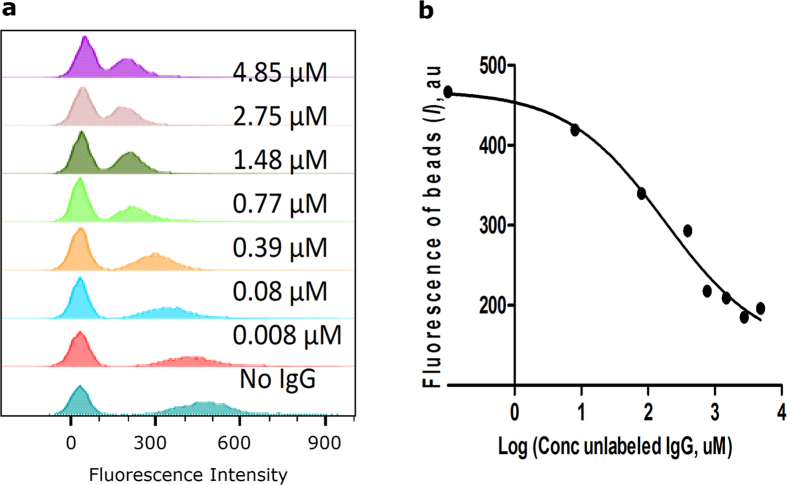
Blocking assay. (**a**) Histograms of beads incubated with unlabelled IgG at shown concentrations and subsequently treated with the fluorescently labelled antibody IR650-IgG (2.5 μM). (**b**) Fluorescence of the beads (*I*) calculated as the Peak Mean Distance (eq. 3) from the histogram analysis: IC_50_ = 0.18 μM for 25 μL of beads, R^2^ = 0.982.

In this work, we postulated that the fluorescence of the bead is proportional to the number of fluorophores, and that the maximum fluorescence comes from the saturation of the beads surface. For that we incubated a standard number of beads with varied quantities of labelled antibodies for each of the studied dyes. To assess the sensitivity of the method and identify the range of concentrations that can be measured, we utilized a blocking method in which the surface of the bead was blocked by a progressively larger concentration of non-labelled antibody, after which it was treated with an excess of a single concentration of fluorescently tagged antibody. The flow cytometry histogram in the corresponding fluorescence channel presents two peaks (Fig. 3). The peaks overlap at high concentrations of unlabelled antibody and become more separated when concentrations are low or no unlabelled antibody are used.

The attempts to reach the full blockade, however, were not successful apparently due to non-covalent attachment of the antibodies to the bead and equilibrium between the competing species (i.e. antibody and dye-antibody conjugate). The shift in the fluorescence negative population observed in this and other experiments is due to some non-specific affinity of the negative beads to antibodies.

This blocking approach enables us to evaluate the span of concentrations to be used for the measurements of the fluorescent brightness. Analysis of the histograms revealed a typical sigmoid curve of the gap between means vs. concentration of the unlabelled IgG (Fig. 3). The 50% inhibitory concentration (IC_50_) was calculated from the nonlinear regression model of *log(inhibitor) vs. response - variable slope*, and was found to be 0.18 μM. Given that the range of concentration typically covers two log units (100-fold change in μM concentration) times the IC_50_, the range of useful concentrations for the given number of beads lies within 0.02–2 μM. It should be noted that the span of concentrations can be different for the beads of different origin, size, or coating.

**Figure 4:**
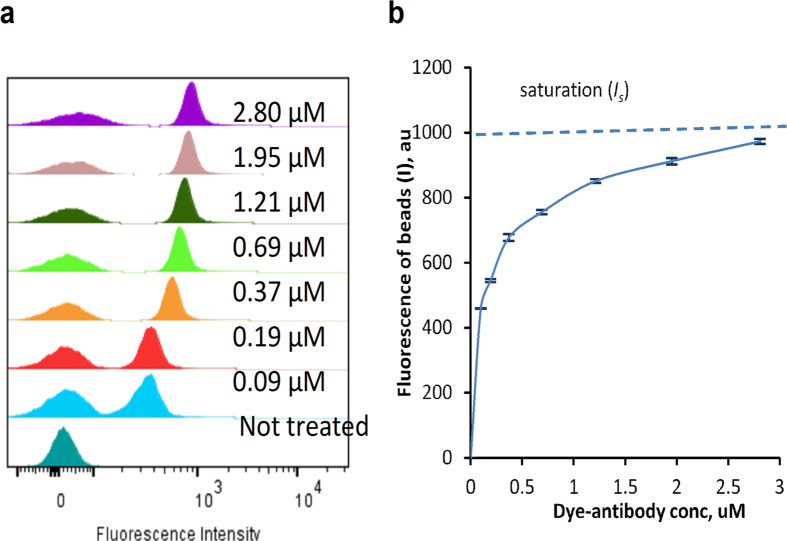
Flow cytometry of beads treated with the IR650-IgG. (**a**) Histograms of beads incubated with IR650-IgG at different concentrations. (**b**) Fluorescence of the beads (*I*) calculated as the Peak Mean Distance (eq. S1–S2, Supplementary Information). The trendline corresponds to the average values of *I*, dashed line shows the saturation level (*I*_*s*_) (averaged, n = 4, error bars represent standard deviation).

The application of our method for fluorescent assessment of IR650-IgG conjugate is illustrated in Fig. 4. Based on the blocking assay and further assay optimization, the concentration of the conjugate varied from 0.03 to 3.0 μM. Increasing the concentration of the dye-conjugate while keeping the quantities of beads fixed predictably increased fluorescence intensity of the beads (Fig. 4a).

The plateau of the fluorescence of beads at higher concentration was due to saturation of the affinity sites on the beads. Fluorescence intensity at the saturation point (*I*_*s*_) (Fig. 4b) corresponds to the fluorescence brightness of the antibody under the excitation/emission conditions specified in the Methods section. Given that 10,000 beads is sufficient for statistical analysis, only 2 μL of beads and less than 10 μg of the dye-antibody are required, making the method highly sensitive.

The reliability and consistency of the measurements were first assessed using a single batch of labelled antibody bound to compensation beads at different concentrations of the conjugates and the fixed amount of the beads. High reproducibility of the results (low standard deviation) is demonstrated in Fig. 4b and suggests low instrumentation error. The reproducibility of the labelling procedure was predictably lower as evidenced by larger standard deviations of the measured *I* value (Figure S4, Supplementary Information).

The presence of multiple fluorophores (DOL > 3) in close proximity can decrease fluorescence brightness of the labelled antibody^22^. Self-aggregation, mostly from *π*-stacking, leads to efficient energy transfer between the dyes, resulting in severe quenching and lowered brightness of the targeted probe^23^. On the other hand, a very low DOL (<0.5) negatively affects fluorescence intensity and may create a significant proportion of unlabelled antibodies^24^. These two factors pose a considerable challenge in preparing sufficiently bright molecular probes.

Many fluorescent dyes are prone to self-quenching when many of them are attached to a protein, an antibody or a nanoparticle^25^. For this reason, having low DOL is a common practice to minimize quenching. However, even at low dye-to-protein ratios, the self-aggregation of the dye becomes substantial due to preferential labelling of the neighbouring residues caused by a self-assembly of the dyes at the surface of the substrate. This results in a clustering of the dye molecules, significant quenching of the fluorescence, and low brightness of the imaging probe. To address this issue, several groups^23^,^26^ proposed modified dyes to decrease the dye-to-dye interactions and increase the brightness of the conjugate. For example, asymmetrical charge prevents the chromophores from *π*-stacking^26^, thus minimizing energy transfer and fluorescence quenching, as well as allowing more fluorophores to be placed on the antibody without sacrificing the brightness.

**Figure 5:**
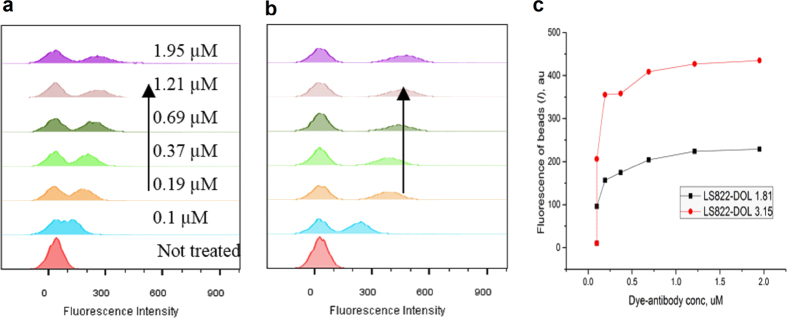
Comparison of the brightness of the beads with LS822-IgG conjugate at different DOL. (**a**) DOL = 1.81. (**b**) DOL = 3.15. Arrows show an increase in dye-conjugate concentration (**c**) Peak mean distance (*I*) comparison between two DOLs.

We used the proposed method to test how the DOL levels affect the brightness. LS822 has an asymmetric charge distribution and is highly hydrophilic. Its hydrophilicity is guaranteed by the three sulfonate groups and ensures the absence of non-specific binding to the beads. The more fluorescently labelled antibody, with a DOL 3.15 (Fig. 5b) vs. 1.81 (Fig. 5a), showed proportionately higher intensity values at equal concentrations of conjugate (as shown in Fig. 5c), suggesting minimal to weak quenching. In contrast, higher DOL in IR650-IgG conjugates did not lead to the additional brightness of the conjugate as judged by the decreased level of the beads fluorescence per DOL (Figure S5, Supplementary Information).

**Figure 6:**
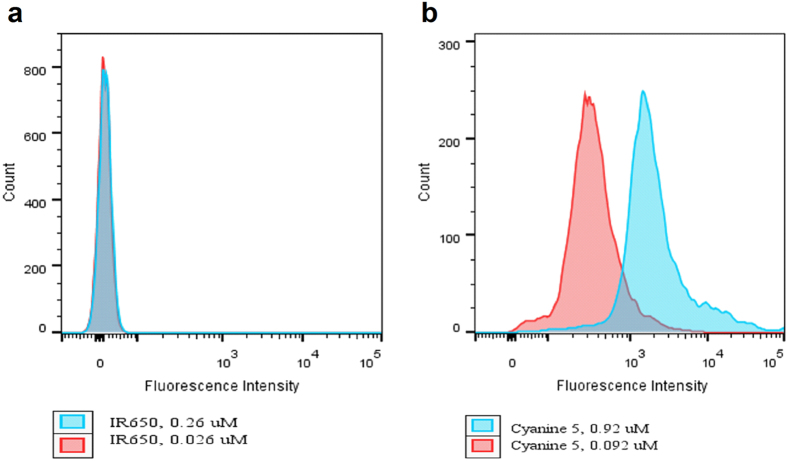
Effect of dye hydrophilicity on binding to beads. (**a**) Free dye IR650, the beads remain unstained, (**b**) Free dye Cyanine 5, shows strong staining of beads including negative population.

Dye-antibody conjugates are frequently contaminated with free unreacted dye. It is expected that free dyes will have negligible affinity to the beads, and therefore no fluorescence will occur in any of the bead populations when incubated with free dye alone. Indeed, when IR-650 and LS822 were incubated with the beads at two concentrations, the histogram showed only a single peak with near zero fluorescence intensity (Fig. 6a) because of the inability of the beads to capture these highly hydrophilic dyes. In contrast, a hydrophobic dye, such as Cyanine 5, showed non-specific and dose-dependent binding to both bead populations (Fig. 6b). This experiment outlines the limitation of the presented method to mostly hydrophilic dyes and their conjugates.

## Conclusions and Considerations

Quantitative measurement of the fluorescent properties of labelled antibodies is necessary for reproducibility in a variety of laboratory assays. It is critical for *in vivo* imaging studies in which low fluorescence demands higher doses of the antibody in order to reach the desired signal-to-noise ratio. We developed a rapid method for measuring the brightness-related parameter of fluorescently-tagged antibodies using a mixture of antibody-capturing positive and negative beads as an internal reference and flow cytometry.

Overall, the procedure requires less than 10 μg of antibody and takes only a few minutes of mixing the fluorescent antibodies with the beads, and then analysing the resulting mixture with a standard flow cytometer. The method is highly reproducible and is applicable for optimizing synthetic conjugation procedures, testing commercial antibodies, and performing high-throughput validation of labelling.

Several considerations have to be taken into account. Different clones of monoclonal antibodies (mAbs) may have different affinity for their corresponding native antigen on the cell surface. Therefore, the brightness of different mAb clones captured on the beads may not be the same as the brightness of the cells stained with the same clones. For brightness comparison of commercially available conjugates directed to the same antigen but of a different clone, Stain Index obtained directly from the cell staining is the valid methodology. Special care should be taken interpreting of conjugate brightness based on beads derived data. Selection of conjugates takes place through the interaction of the kappa light chain of the antibody with the anti-kappa antibody on the surface of the bead. The same conjugate, however, interacts with cell antigen through the antigen-binding domain, which is different from a kappa-light chain of an antibody. Given that flow cytometry measurements depend on a number of instrumentation-related parameters, the presented method only provides the relative brightness and cannot determine DOL directly. Absolute values of the brightness will require a set of recognized standards, for example, beads with known fluorescent brightness. The second consideration is that the imaging optics for *in vivo* studies are, in general, different from standard flow cytometry configurations (excitation lines, emission filters). Thus, care has to be taken for direct comparison of conjugates with respect to their performance under imaging conditions. Flow cytometers with dedicated “imaging-like” channels should be preferred for the best translatability between the techniques. Finally, the beads with lower non-specific affinity and beads covered with specific antigen instead of broad-spectrum activity would allow for more universal applicability of the method.

NHS ester conjugation, so far the most popular method for antibody labelling, leads to variability in the degree of labelling and therefore, to the broad distribution of brightness among fluorescently tagged antibodies. The proposed method can potentially address this question and characterize the variability of labelling by calculating the standard deviation (σ_2_) of the signal from the positive beads. For that, the beads should have relatively low coverage to minimize the averaging. Although we do not present this analysis, the described approach can be used to optimize the conjugation process and establish uniformly labelled conjugates. The method can be also applied to compare alternative labelling techniques, such as utilizing maleimide coupling to sulfhydryls, or through conjugation to the carbohydrate via reductive amination.

## Methods

### Materials

Polystyrene beads (OneComp eBeads, known as compensation beads) were purchased from eBioscience. Each sample of beads contains a positive and negative population. The positive population is coated to have high affinity for kappa chains of antibodies and captures mouse, rat, and hamster antibodies. The negative uncoated population of beads is unreactive, with no sites to capture antibodies. This negative population was expected not to show fluorescence after treatment with the dye-antibody conjugate and hence was used an internal reference. The concentration of beads is 2.5 × 10^5^ per drop (50 μL) in 0.1% BSA/PBS buffer stabilized with 0.09% sodium azide. IRDye 650 NHS Ester was obtained from LI-COR, Cyanine5-NHS ester was purchased from Lumiprobe. Immunoglobulin G (IgG) from rat serum, reagent grade, lyophilized powder, cat. I4131 was purchased from Sigma-Aldrich.

A dye LS822 and the conjugates of IR650-IgG and LS822-IgG were synthesized, purified, and characterized as specified in the Supplementary Information. The structures of the NHS activatable dyes used in this study are shown in Fig. 2.

### Dye-to-antibody (D/P) ratio

The molar absorptivity coefficient of the antibodies at 280 nm (*ε*_*p,280*_) was set to 190,000 M^−1^cm^−1^ for IgG according to the manufacturer. The molar absorptivities for the dyes in water were determined to be 130,000_ _M^−1^cm^−1^ for LS822 using Beckman Coulter DU-640 UV-vis spectrophotometer, and 230,000_ _M^−1^cm^−1^ for IR650 as reported by the manufacturer. The D/P ratio of the bioconjugates was calculated according to a known eq. 2^23^,^27^.


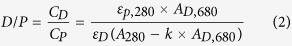


Where A_280_ is the absorbance of the IgG-dye conjugate at 280 nm and A_D,680_ is the absorbance of the conjugate at 680 nm. The absorbance of the dye at 280 nm was corrected by the factor *k* = *A*_*D,280*_/*A*_*D,680*_.

### Beads-sample preparation

Purified dye-antibody conjugates or free dyes in PBS buffer (0.5, 1, 2, 4, 8, 16 and 32 μL) were mixed with 25 μL of compensation beads at 4 °C for 30 minutes. Samples were centrifuged at 3,000 rpm for 5 min at 4 °C. Supernatant was removed and the beads were washed once, resuspended in 50 μL PBS, and diluted to 500 μL PBS.

### Blocking assay

Varying amounts (0.01, 0.1, 0.5, 1, 2, 4, and 8 μL) of unlabelled 20 μM IgG were added to 25 μL compensation beads, and incubated at 4 °C for 30 min. Beads were then isolated by centrifugation, washed once with PBS, and resuspended in an excess (30 μL) of IgG-IR650 conjugate, with the final concentration at 2.5 μM. Beads were further resuspended in 50 μL PBS and diluted to 500 μL PBS prior to analysis by flow cytometry. IC_50_ was calculated from the resulting *I* values according to the eq. 3 implemented in Prizm 5.0 (GraphPad Software, Inc.).


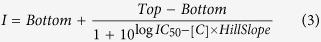


Where *I* is the gap between peaks, *Top* and *Bottom* are plateaus in arbitrary units, [*C*] is the concentration of unlabeled IgG in nM, *IC*_*50*_ is the concentration of unlabeled antibody in nM, and *HillSlope* is the steepness of the curve.

### Treatment of beads with free dyes

A solution (3 μL) of either IR650 (0.175 mM) or Cyanine 5 (0.6 mM) in DMF were mixed with 75 μL of PBS buffer. The resulting solutions (0.1 μL and 1.0 μL) were mixed with 25 μL beads each. Final concentrations were 0.026 μM and 0.26 μM for IR650, 0.092 and 0.92 μM for Cyanine 5. The beads were separated from the solution by centrifugation, the supernatant removed and beads further re-suspended in 50 μL PBS diluted to 500 μL PBS prior to the analysis by flow cytometry.

### Microscopy of beads

For microscopy studies, beads were incubated with a solution of 10 μL of the conjugate in 250 μL of PBS. Before and after the treatment, the beads (1 μL) were placed on a glass slide, covered with a coverslip, and sealed with a nail polisher. Differential image contrast (DIC) and fluorescent images of the beads were recorded with a BX51 Olympus Microscope equipped with Nomarski optics for objective 40X and a Cy5.5 filtercube (Semrok). Images were captured with a Lumenera 3 camera using Infinity Analyze 6.0 software.

### Flow Cytometry

Flow cytometry experiments were performed using a multichannel flow cytometer (Beckman Coulter). The results were processed with *FlowJo X* software package and custom made MATLAB-based software *Label_It* developed in our lab. The background fluorescence was eliminated by adjusting the gain of the corresponding photomultiplier tubes (PMTs) in the flow cytometer. The scale was set by adjusting the position of the maximum of non-treated beads to zero. Samples of the unconjugated (unlabelled) and saturated beads were run to set the gain of the PMT at the relevant channel. An argon ion laser (637 nm) and a 660/20-nm bandpass filter were used for fluorescence measurements.

### Histogram analysis

**Figure 7:**
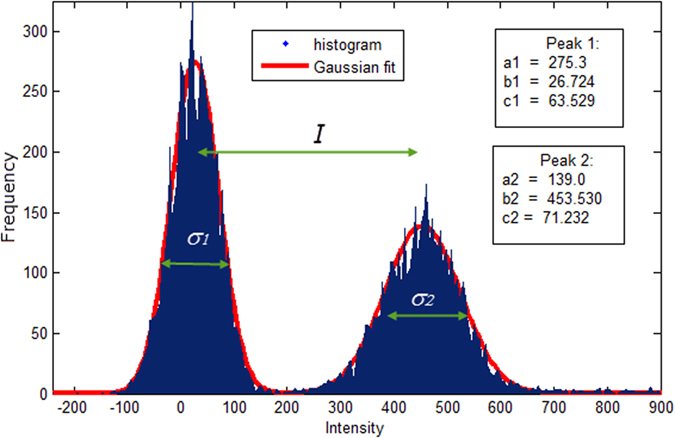
Example of fitting the bimodal Gaussian distribution model to the flow cytometry histogram from beads treated with fluorescent antibodies. R^2^ = 0.983. *I* = *b*_*2*_*-b*_*1*_, σ_1_ and σ_2_ - standard deviations of negative and positive peaks, calculated as 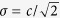.

The brightness-related parameters of the antibodies were calculated using *Label_It* software by fitting a histogram (Fig. 7) with a two-member Gaussian distribution model, (eq. S1, Supplementary Information).

The goodness of fit was judged by visual observations, residual plots, R-square value, which typically exceeded 0.96, and RMSE. The output provided the heights, means, and widths for each peak: where the parameter *a* is the height of the curve’s peak, *b* is the position of the centre of the peak (mean), and *c* is the width of the peak. We used established metrics for evaluation of bimodal histograms based on fitting of the histogram distribution. This include Peak Mean Distance (*I*), defined as a gap between two peaks, Stain Index (*SI*)^19^, and Bhattacharya distance (*D*_*b*_)^28^ (Calculated using the equations eq. S2–S4, Supplementary Information).

The limits of fluorescence (usually within −200 to 1200) and bins width (usually 20) were set automatically. The two parameters can be also adjusted to obtain the best fit as judged by R-square or RSME values. Figure S6, Supplementary Information, illustrates the effect of the bins width on the RMSE, *I, SI* and *D*_*B*_. We found that for a typical 10,000 events, the value 5 < N < 20 provides the best fit across a variety of dye-conjugates. We also found that *I* provides a more stable and predictable metric compared to *SI* or *D*_*B*_(Figure S7, Supplementary Information).

We compared *I, SI, D^B^* and found *I* is preferable. The difference between *I* and other metrics such as *SI* and *D^B^*, is that the latter take into account standard deviations. These standard deviations are calculated from fitting of the flow cytometry histogram with a bimodal Gaussian-type distribution. Although this fitting provides appropriate R-square values (>0.9), it is not very accurate at highly overlapped or well separated peaks. Parameter *I* is less dependent from standard deviation and appears to be more robust with lower variability (such as shown in Fig. 4b).

## Additional Information

**How to cite this article:** Zhou, H. *et al*. Cell-free measurements of brightness of fluorescently labeled antibodies. *Sci. Rep.* **7**, 41819; doi: 10.1038/srep41819 (2017).

**Publisher's note:** Springer Nature remains neutral with regard to jurisdictional claims in published maps and institutional affiliations.

## Supplementary Material

Supplementary Information
