# Supplementary material for: Cell-free measurements of brightness of fluorescently labeled antibodies
Source: Sci Rep. 2017 Feb 2;7:41819. doi: 10.1038/srep41819 (PMC5288790; doi:10.1038/srep41819)
Supplement: Supplementary Information [file srep41819-s1.pdf]

Supplementary Information

Cell-free measurements of brightness of fluorescently labeled antibodies

Haiying Zhou,[a‡] George Tourkakis,[a‡] Dennis Shi,[a‡] David M. Kim,[a] Hairong Zhang,[a] Tommy Du,[a] William C. Eades,[b] Mikhail Y. Berezin[a*]

[a]Department of Radiology, [b]Department of Medicine and Siteman Cancer Center Flow Cytometry Core, Washington University School of Medicine, St. Louis, MO 63110

1. EXPERIMENTAL PART

1.1. Materials

High-purity water (18.2 MΩ) was used throughout the study. Organic solvents and reagents were acquired from Thermo Fisher Scientific and Sigma-Aldrich and used unpurified. Polystyrene beads (OneComp eBeads) were purchased from eBioscience. Each sample of beads contains a positive and negative population; the positive population captures mouse, rat, and hamster antibodies, and the negative population is unreactive. The concentration of beads is 2.5 x 10^5 per drop (50 uL) or 5,000 beads/uL in 0.1% BSA/PBS buffer stabilized with 0.09% azide. IRDye 650 NHS Ester was obtained from LI-COR, Cyanine5-NHS ester was purchased from Lumiprobe. immunoglobulin G (IgG) was purchased from Sigma-Aldrich. A dye LS822 and the conjugates of IR650-IgG and LS822- IgG were synthesized, purified, and characterized as specified below.

1.2. Instrument and characterization

General: ^{1}H NMR data were recorded on a 400 MHz Varian spectrometer at ambient temperature in either chloroform-*d* or methanol-d_4 and referenced to tetramethylsilane (TMS) as an internal standard unless otherwise stated. Chromatographic purification was performed on a C18 silica gel column. LC-MS spectra were recorded on a Shimadzu 2010A (ESI, positive mode) equipped with UV/Vis detector with up to 900 nm detection limit. Thin layer chromatography (TLC) was performed on either alumina-backed plates coated with 0.20 mm silica gel Xtra SIL G/UV_{254} or 0.15 mm RP-18W/UV_{254} silica gel.

Optical spectroscopy. The UV-visible spectra of LS822 were recorded at room temperature using an HP 8354 spectrophotometer. The emission was recorded using the experimental setup based on the spectrofluorometer (Horiba) using CCD camera Synapse (Horiba). The excitation was conducted with a Xe-lamp using a double grating (2x1200 gm/mm @ 500 mm) monochromator. Absolute fluorescence quantum yield of the tablet was measured using a 150 mm (6 inch) integrating sphere using a fiber optic bundle connected to the spectrophotometer.

MALDI: The compound was mixed with a 10 mg/mL α-cyano-4-hydroxycinnamic acid (CHCA, Waters (Milford, MA, USA)) matrix in 50% ACN, and 0.1% TFA to give a final concentration of 5 pmol/µL, which was added to Applied Biosystems (AB) V700666 Sample Plate. A VOYAGER DE-PRO Matrix-assisted laser desorption/ionization (MALDI)-mass spectrometry (MS) from AB

was used for the study. The instrument employs a time-of-flight mass analyzer and a nitrogen laser that operates at 337 nm to ionize the sample. The MS function in reflector modes for positive ions were applied. The MALDI-MS spectral data were processed using the Data Explorer software from AB.

SDS: SDS agarose gel protein electrophoresis was conducted using a gel Certified™ Molecular Biology Agarose and 10x Tris/Tricine/SDS buffer according to manufacturer's protocol (Bio-Rad Laboratories). The gel was imaged using a home-made imaging system[3] equipped with a deep cooled CCD camera Versarray (Ropper), a SWIRON 1.4/23 Compact (Schneider) lens, 660 nm mounted variable power LED and 700 long pass filter (Thorlabs). The images were acquired with WinView 32 software and processed with ImageJ.

Due to its low molecular weight and overall negative charge, free dye appears at the bottom of the gel, while the fluorescent-labeled antibody appears in the middle of the gel as a single band, indicating successful conjugation.

1.3. Synthesis

IR650-IgG conjugate: IgG (0.75 mg) was dissolved in 300 µL of 50 mM $NaHCO_3$ buffer and mixed with varying amounts of dye from a stock solution of IR650 (0.1 mg) in 5 µL DMSO. The reaction was shaken at room temperature for 2 h. The conjugate was purified on a Sephadex G-25 column with 1x PBS buffer. Blue colored fractions were collected (total 1.0 mL). Fractions were evaluated using SDS agarose gel protein electrophoresis to confirm conjugation. Expected final concentration of the dye-conjugate was 1.5 mg/mL (5 µM).

LS822: The dye was synthesized similar to our previously published procedure for asymmetrical cyanine dyes.[1] Briefly, in a vial equipped with a stir bar, a known indolenine **1** (213.3 mg, 0.53 mmol) and malonaldehyde bis(phenylimine) monohydrochloride (2) (Sigma-Aldrich) (207.30 mg, 0.80 mmol) were added to a mixture of Ac_2O (3.2 mL) and AcOH (3.2 mL) and heated to reflux for 4 h. The progress of the reaction was monitored by thin layer chromatography (TLC, C18, eluent H_2O-MeOH 1:1, R_f = 0.83 for **3**,) and UV-Vis (by disappearance of the absorption peak at 385 nm and the appearance of a strong peak at 456 nm). Acetic acid was removed with a rotary evaporator and the product was triturated by EtOAc to remove excess of **2**. The crude intermediate **3** was used in the next step without further purification. The intermediate **3** (171.1 mg, 0.35 mmol) was dissolved in acetic anhydride (3.35 mL) and pyridine (3.35 mL) mixture. The UV spectrum showed an absorbance at 454 nm for the acetate form of the intermediate. A known carboxy-indolenium **4**[2] was added and the reaction mixture was heated to 110 ^oC and stirred for 10 min until the reaction mixture turned blue. The reaction was monitored by the disappearance of the absorption peak at 454 nm and the appearance of a strong peak at 651 nm. The mixture was cooled to room temperature and the dye was precipitated by addition of EtOAc. The gummy residue was washed with EtOAc and 2-propanol. The product was dried under reduced pressure to afford LS822 as a blue solid (192.4 mg, yield 76%). TLC (C18, H_2O-MeOH 1:1, R_f = 0.75). ESI-MS *m/z*: 723.83 [M^+]. ^1H NMR (400 MHz, CD_3OD) δ 9.70 (d, J=4), 9.04 (d, J=4), 8.35 (d, J=8), 8.34-8.24 (m), 8.24-8.08 (m), 7.91 (d, J=8), 7.51 (d, J=8), 7.46 (d, J=8), 4.85 (d, J=8), 4.43-4.31 (m), 2.86 (d, J=8), 2.32-2.21 (m), 1.80 (s), 1.76 (s). Absolute quantum yield in 1xPBS buffer: 21.79%, in DMSO 53.36%. Molar absorptivity in PBS: 130,000 $M^{-1}cm^{-1}$

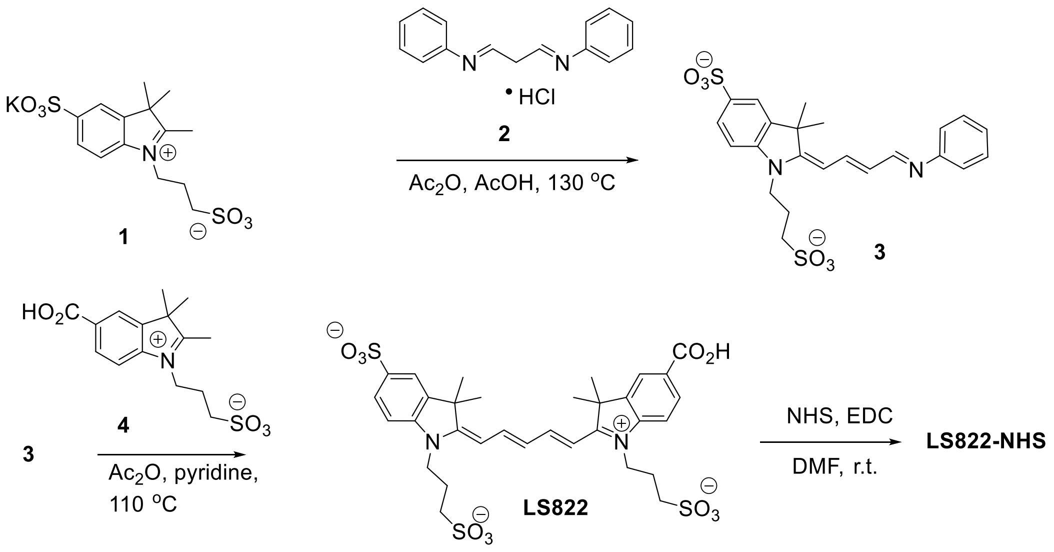

Scheme 1 Synthesis of LS822 dye

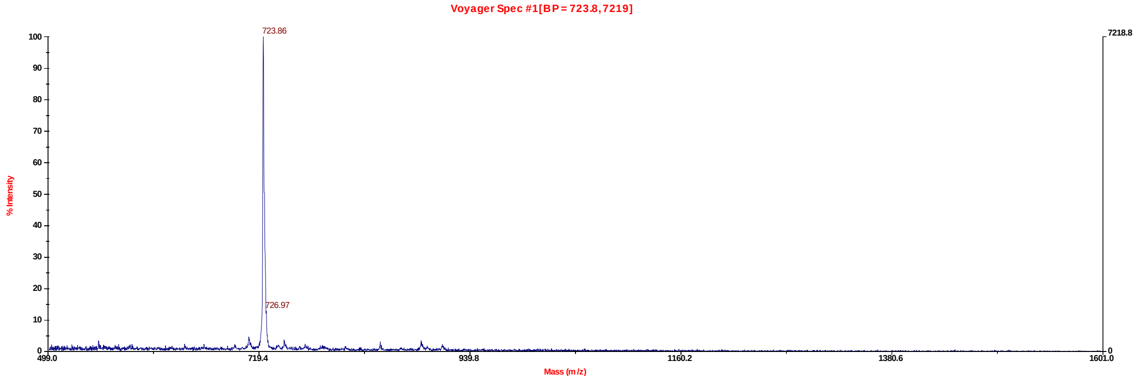

LS822 analyzed by ABI 4700 MALDI TOF-TOF, Found: 723.86, calculated: 723.85

LS822-NHS: Activated LS822-NHS-ester was synthesized similarly to our previously published procedure[1]. Briefly, to an 8 mL vial equipped with a stir bar, LS822 (49 mg, 0.068 mmol) was added and dissolved in dry DMF (2 mL). N-hydroxysuccinimide (NHS) (23.4 mg, 0.204 mmol) and 1-ethyl-3-(3-dimethylaminopropyl)-carbodiimide (EDC) (39.0 mg, 0.204 mmol) were added and the reaction mixture was stirred overnight at room temperature. The reaction was monitored by TLC for the production of LS822-NHS. $R_f = 0.25$ (C18, H_2O-ACN 75:25). Diethyl ether was added to the reaction mixture to precipitate the product. The obtained ester was re-dissolved in a minimal amount of methanol and triturated with diethyl ether. This step was repeated three times to give the desired NHS-ester as a blue solid (40 mg, yield 72%). ESI-MS *m/z* 820.93 [M].

LS822-IgG-conjugate: IgG (0.75 mg) was dissolved in 250 µL of 50 mM $NaHCO_3$ buffer and mixed with varying amounts of dye from a solution of LS822 (0.1 mg) in 10 µL of DMF. The reaction was left shaking at room temperature for 2 h. The conjugate was purified on a Sephadex G-25 column with 1x PBS buffer. Blue colored fractions were collected (total 1.0 mL). Fractions were evaluated using SDS agarose gel protein electrophoresis to confirm conjugation. Expected final concentration of the dye-conjugate was 1.5 mg/mL (5 µM).

1.4. Brightness-related parameters from flow cytometry

The brightness-related parameters of the antibodies were calculated using *Label_It* software by fitting a histogram with a two-member Gaussian distribution model (eq. S2). Gaussian bimodal fitting was efficient in modelling the distribution of the histogram even for highly overlapped peaks (such as shown in Figure S1).

$$f(x) = a_1 \exp\left[-\frac{(x-b_1)^2}{c_1^2}\right] + a_2 \exp\left[-\frac{(x-b_2)^2}{c_2^2}\right] \quad \text{(S1)}$$

where the parameter *a* is the height of the curve's peak, *b* is the position of the center of the peak (mean), and *c* is the width of the peak.

Peak Mean Distance (*I*) defined as a gap between two peaks, Stain Index (*SI*)[3], and Bhattacharya distance (D_b)[4] were calculated using the following equations:

$$I = b_2 - b_1 \quad \text{(S2)}$$

$$SI = (b_2 - b_1)/2\sigma_1 \quad \text{(S3)}$$

$$D_B = \frac{1}{4}\ln\left[\frac{1}{4}\left(\frac{\sigma_1^2}{\sigma_2^2} + \frac{\sigma_2^2}{\sigma_1^2} + 2\right)\right] + \frac{1}{4}\left[\frac{(b_2 - b_1)^2}{\sigma_1^2 + \sigma_2^2}\right] \quad \text{(S4)}$$

Where σ_1 and σ_2 - standard deviations of negative and positive peaks, calculated as $\sigma = c/\sqrt{2}$

2. FIGURES

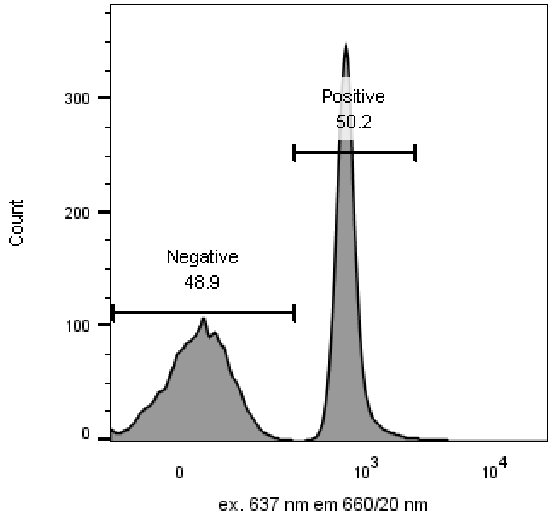

Figure S1 Number of negative and positive beads is approximately 1:1.

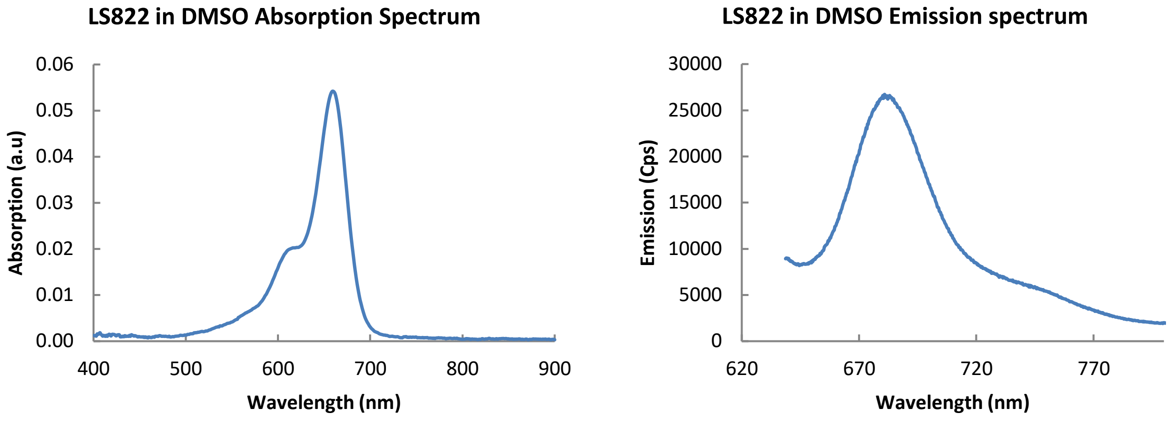

Figure S2 Optical spectra of LS822 in DMSO (excitation 625 nm)

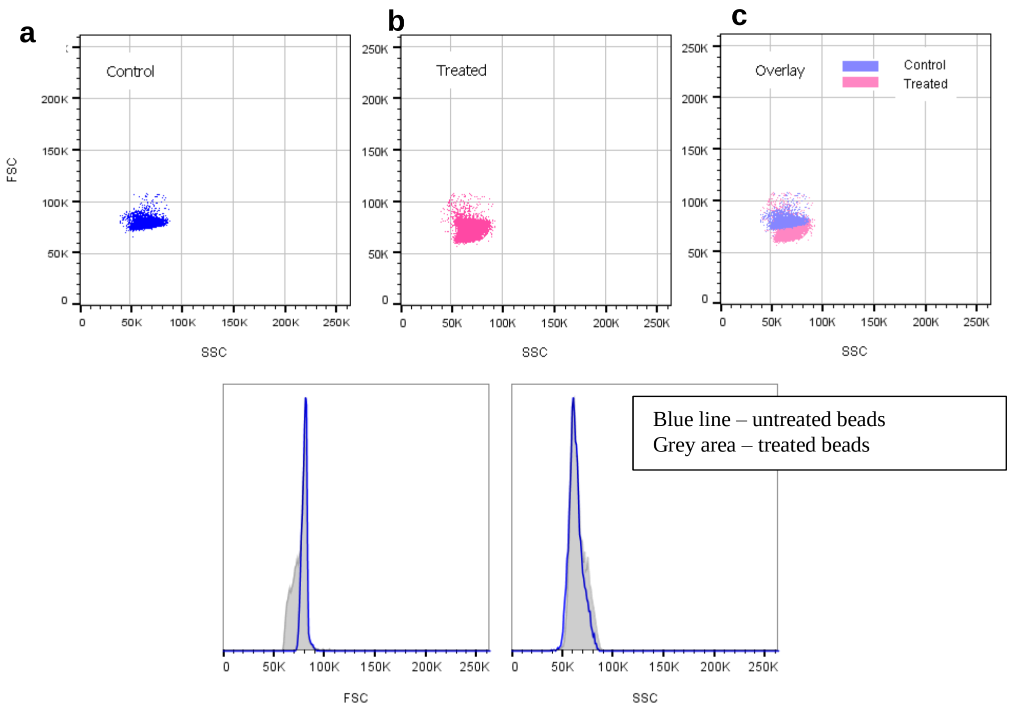

Figure S3 Flow cytometry of the beads untreated and treated with the fluorescently labeled antibodies. Upper panel: Side scattering (SSC) vs. front scattering (FSC). (**a**) Control, untreated beads, **(b)** Beads treated with IR650-IgG (2.0 μM). **(c)** Overlap. Low panel shows corresponding histogram for FSC and SSC. Almost identical scattering properties (as judged by low <5% indicate beads stability towards the treatment, Overton cumulative subtraction algorithm)

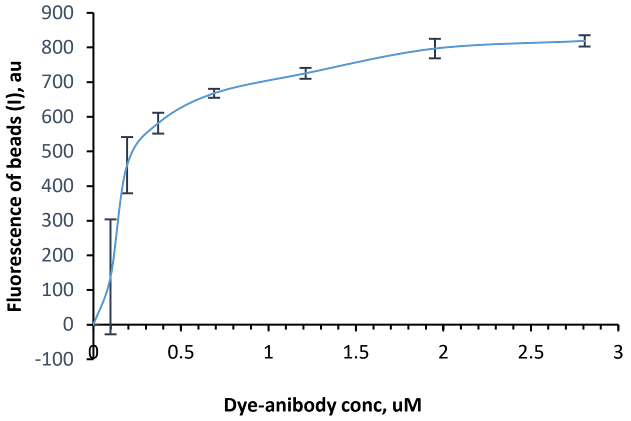

Figure S4 Evaluation of conjugation reproducibility. Different batches of IgG-IR650 were synthesized separately using identical procedures. Fluorescence of the beads calculated as the gap between peaks (*I*). The trendline corresponds to the average *I* values, error bars show standard deviation (n=4). Note: the error is larger at low conjugate-to-bead ratio (low concentration of the conjugate) and become close to the instrumentation error at the beads saturation level (I_s).

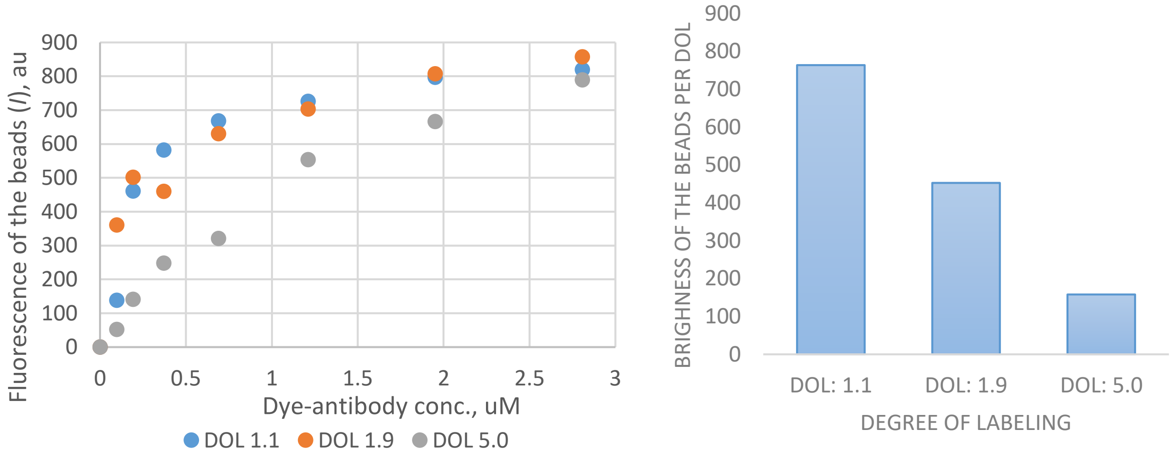

Figure S5 Effect of the degree of labeling for IR650-IgG conjugates. A: Change in fluorescent brightness of the beads for different conjugates. B: Normalized brightness of the beads measured at the saturation level per DOL.

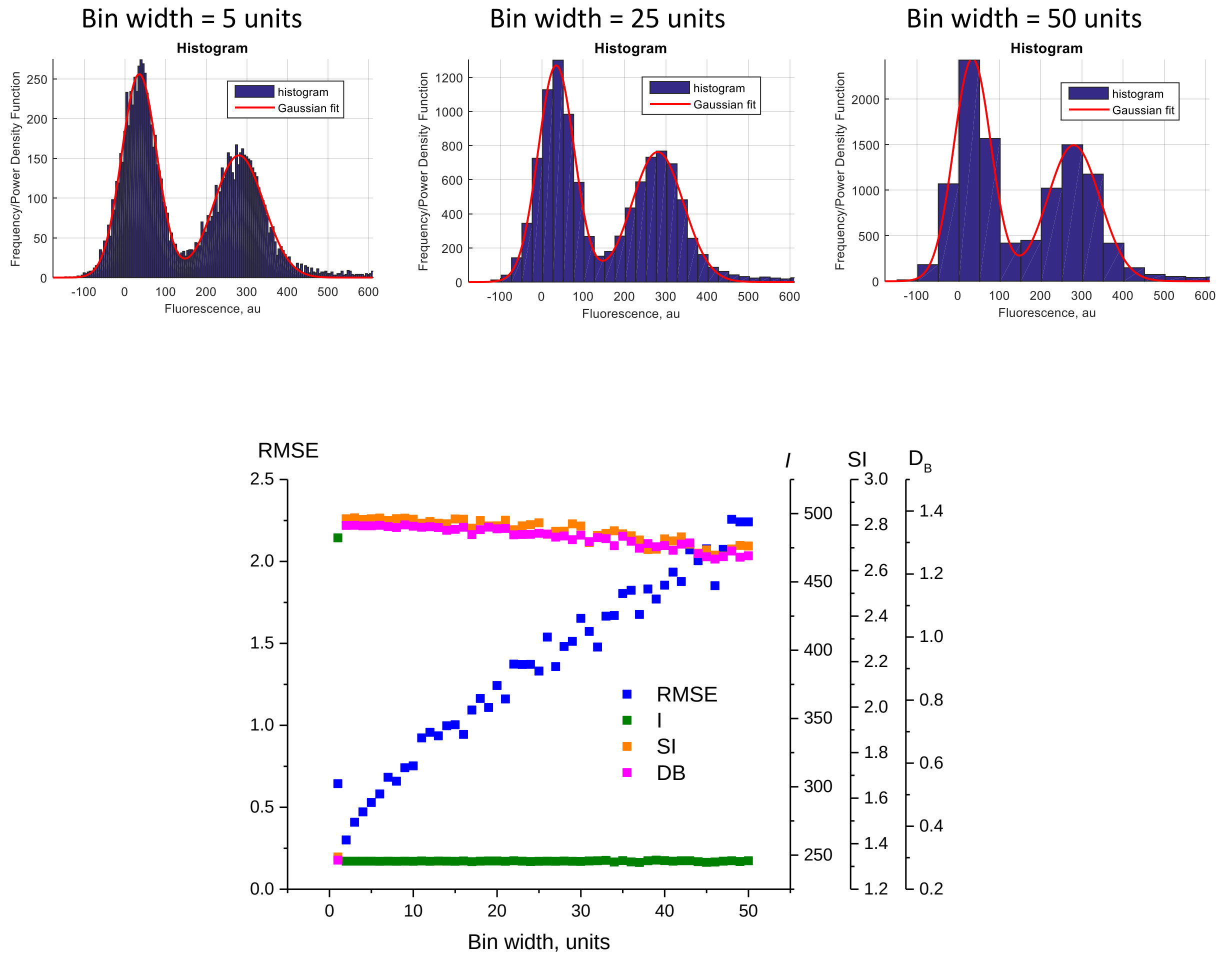

Figure S6 Effect of the bin width on the quality of the fit and calculated parameters. Top panel: Histograms of the same sample at different bin widths: N=5, 25, and 50. Low panel: effect of the bin width on RMSE as the quality of the fit, Gap Between Peaks (*I*), Stain Index (*SI*) and Bhattacharya Distance (D_B). At very low and very high bin width RMSE values become large and the fit becomes not reliable.

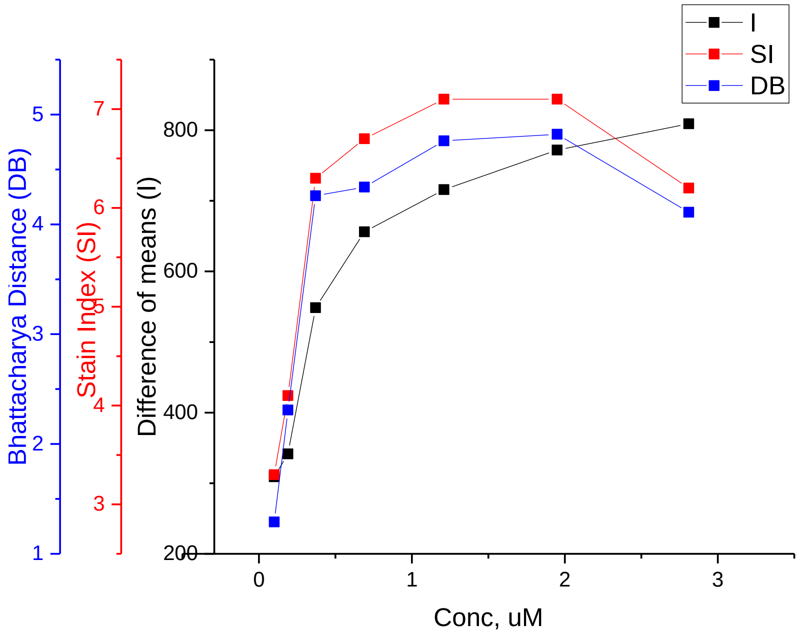

Figure S7 Comparison of the different metrics in evaluation of IR650-IgG conjugates. Peak Means Distance (calculated as a difference of means or the gap between peaks) was found to be the most stable parameter.

REFERENCES

1 Zhegalova, N. G., He, S., Zhou, H., Kim, D. M. & Berezin, M. Y. Minimization of self-quenching fluorescence on dyes conjugated to biomolecules with multiple labeling sites via asymmetrically charged NIR fluorophores. *Contrast Media Mol Imaging* **9**, 355-362,

2 Gustafson, T. P., Cao, Q., Achilefu, S. & Berezin, M. Y. Defining a polymethine dye for fluorescence anisotropy applications in the near-infrared spectral range. *Chemphyschem* **13**, 716-723,

3 Maecker, H. T., Frey, T., Nomura, L. E. & Trotter, J. Selecting fluorochrome conjugates for maximum sensitivity. *Cytometry Part A* **62A**, 169-173,

4 Kim, D. M. *et al.* Highly sensitive image-derived indices of water-stressed plants using hyperspectral imaging in SWIR and histogram analysis. *Sci Rep* **5**, 15919,
